# Determination of Rate and Causes of Wastage of Blood and Blood Products in Iranian Hospitals

**DOI:** 10.4274/tjh.2012.0105

**Published:** 2014-06-10

**Authors:** Rafat Mohebbi Far, Fatemeh Samiee Rad, Zahra Abdolazimi, Mohamad Mehdi Daneshi Kohan

**Affiliations:** 1 Qazvin University of Medical Sciences, Department of Health Management, Qazvin, Iran; 2 Qazvin University of Medical Sciences, Metabolic Research Center, Department of Pathology, Qazvin, Iran; 3 Master of Health Management; 4 Qazvin University of Medical Sciences, Department of Laboratory, Qazvin, Iran

**Keywords:** Blood, Blood component, Wastage, Transfusion, Blood bank

## Abstract

**Objective:** The purpose of this study was to determine the rate and causes of wastage of blood and blood products (packed red cells, plasma, platelets, and cryoprecipitate) in Qazvin hospitals.

**Materials and Methods:** The study was conducted in all hospitals in Qazvin, including 5 teaching hospitals, 2 social welfare hospitals, 3 private hospitals, 1 charity hospital, and 1 military hospital. This descriptive study was based on available data from hospital blood banks in the province of Qazvin. The research instrument was a 2-part questionnaire. The first part was related to demographic characteristics of hospitals and the second part elicited information about blood and blood component wastage. The collected data were then analyzed using descriptive statistic methods and SPSS 11.5.

**Results:** Blood wastage may occur for a number of reasons, including time expiry, wasted imports, blood medically or surgically ordered but not used, stock time expired, hemolysis, or miscellaneous reasons. Data indicated that approximately 77.9% of wasted pack cell units were wasted for the reason of time expiry. Pack cell wastage in hospitals is reported to range from 1.93% to 30.7%. Wastage at all hospitals averaged 9.8% among 30.913 issued blood products. Overall blood and blood product (packed red cells, plasma, platelets, and cryoprecipitate) wastage was 3048 units and average total wastage per participant hospital for all blood groups was 254 units per year.

**Conclusion:** Blood transfusion is an essential part of patient care. The blood transfusion system has made significant advancements in areas such as donor management, storage of blood, cross-matching, rational use of blood, and distribution. In order to improve the standards of blood banks and the blood transfusion services in Iran, comprehensive standards have been formulated to ensure better quality control in collection, storage, testing, and distribution of blood and its components for the identified major factors affecting blood product wastage.

## Abstract (tr)

**Amaç:** Bu çalışmanın amacı, Qazvin hastanelerinde kan ve kan ürünleri (eritrosit süspansiyonu, plazma, trombosit ve kriyopresipitat) israfının oran ve nedenlerinin belirlenmesiydi.

**Gereç ve Yöntemler:** Bu çalışma, 5 eğitim hastanesi, 2 sosyal yardım hastanesi, 3 özel hastane, 1 hayır kurumu hastanesi ve 1 askeri hastaneyi içerecek şekilde Qazvin’in tüm hastanelerinde yürütüldü. Çalışmada, Qazvin sınırları dahilindeki hastanelerin kan bankalarından elde edilen veriler temel alındı. Araştırma materyali, iki bölümden oluşan bir anket formuydu. İlk bölüm, hastanelerin demografik özellikleriyle alakalıyken, diğer bölümde ise hastanelerde israf edilen kan ve kan ürünleri hakkında aydınlatıcı bilgiler irdelenmekteydi. Elde edilen veriler, tanımlayıcı istatistiki yöntem ve SPSS 11.5 kullanılarak analiz edildi.

**Bulgular:** Kan israfı, son kullanma tarihinin dolması, cerrahi ya da diğer kliniklerden istem yapılmasına karşın kullanılmaması, raf ömrünün dolması, hemoliz ve çeşitli başka nedenler dahil olmak üzere pek çok nedenden ötürü olabilmektedir. Verilere göre, israf edilen eritrosit süspansiyonlarının %77,9’u son kullanma tarihinin geçmesi nedeniyle kullanılamamıştır. Kan ürünlerinin israfı, hastane verilerine göre %1,93 ile %30,7 arasında değişmektedir. Tüm hastanelerdeki 30,913 kan ürünü için ortalama kan ürünü israfı %9,8 olmuştur. Toplam kan ve kan ürünü (eritrosit süspansiyonu, plazma, trombosit ve kriyopresipitat) israfının 3048 unite, tüm kan grupları için hastane başına yıllık ortalamanın ise 254 ünite olduğu saptanmıştır.

**Sonuç:** Kan transfüzyonu hasta bakımının yaşamsal bir parçasıdır. Kan transfüzyonu disiplini ise verici yönetimi, kan depolanması, cross-match, kanın akılcı kullanımı ve ayrımı gibi alanlar başta olmak üzere pek çok alanda belirgin ilerlemeler kaydetmiştir. İran’daki kan transfüzyonu yapılan servislerin ve kan bankalarının standartlarının geliştirilmesi için, kan ürünleri israfını etkileyen en önemli nedenleri göz önünde tutacak şekilde kan ve kan ürünlerinin toplanması, depolanması, test edilmesi ve dağıtılmasında yüksek kalite kontrolün elde edilmesine yönelik olarak geniş kapsamlı standartlar oturtulmaktadır.

## INTRODUCTION

There has been a continuing need for blood transfusion in recent years. Several factors are involved, including the increase of life expectancy and advances in medical technology [1]. The industrial world and its ever increasing health incidents increase the use of blood and its products. All of these issues have been causing concern over the adequacy of the blood obtained from human sources to meet increasing demands, doubling the importance of consumption management to prevent the loss of such resources [2]. It should be noted that today a very small number of countries have utilized the necessary structures and resources to improve blood transfusion centers [3]. The key elements of success are the use of financial and material resources, preparation and implementation of a realistic plan of action based on actual description, and analysis of the existing conditions in each country, availability of blood, proper and standard storage conditions, proper clinical use of blood in treatments, and compliance with rules and regulations for prescription of blood and its products [4]. However, the number of blood donors during the past few years has been steadily declining. Reasons include the increase of donors’ age, which causes morbidity, or increased and stricter parameters for the acceptance of donors in terms of their health and infectious agents associated with blood transfusion [5]. Greinacher et al. analyzed the effects of demographic changes on the blood supply in eastern Germany as a model region for Europe and found that a decrease of the young donor population will decrease the number of donors by 27.5%-32.6% in 2015. The increasing size of the elderly population will also increase the number of blood transfusions by 11.8%-13.9% due to morbidity increase [6]. A general decline in the number of donors is being seen for the first time and the increasing age of the population limits the number of donors being attracted and retained [7]. To solve this problem, we need to implement a rigorous scientific protocol on blood reserves and proper and timely use of blood products. It seems we can overcome these problems considerably through accurately describing a protocol and creating more coordination, preventing the loss of blood products and energy, human, and financial resources [8]. There has even been talk about blood overuse since 1937, when Fantus wrote: “In this hospital, blood is overly used” [9]. A 1953 study revealed that 50 out of 290 cases of blood transfusion were unnecessary and proposed a training program about the proper use of blood for physicians [10]. In 1961, the Joint Commission on Accreditation of Health Organizations decided to approve hospitals by reviewing their use of blood [11]. In 1991, the AABB declared reviewing of blood used by its blood transfusion committee as mandatory for the first time [12]. In October 1995, the World Health Organization held its first preparatory meeting for the formation of a workgroup of international cooperation for blood safety in Geneva, and it published its recommendations for the creation of national guidelines and policies for the clinical use of blood in 1998 [13]. Today, the importance of blood transfusion medicine is well established, but procuring healthy blood only ensures a part of its safety. The other part is the proper shipping, storage, and use of blood in hospitals (Table 1). Therefore, excessive and inappropriate use of blood products is also among the factors cited; this requires public commitment to ensure that healthy blood and its products are available at reasonable prices, are used properly with minimum losses, and are produced within a consistent health monitoring system [14]. Four main processes that are implemented during blood transfusions in hospitals of Qazvin Province, one of the central provinces of Iran, are: the requests for blood and its products by physicians form the organization of blood transfusions; these products are stored in blood banks of hospitals; compatibility tests are conducted before the injection; and the injection process and its side effects are monitored. Meanwhile, blood wastes occur by various causes that are the focus of this study (Table 2). This study was designed to investigate the amount and causes of waste of blood and its products (packed red cells, plasma, platelets, and cryoprecipitate) in hospitals of Qazvin Province. This study may provide experience-based information to various groups and act as a helpful guideline for optimum use of blood and reduction of its wastes. 

## MATERIALS AND METHODS

This was a cross-sectional, analytical-descriptive study based on the statistical data available from hospitals’ blood banks. Data about blood and blood component wastage for the year 2010 were obtained from 12 hospitals, including 5 teaching hospitals, 3 private hospitals, 2 social welfare hospitals, 1 charity hospital, and 1 military hospital. The hospitals were divided into wards according to type and complexity of activities undertaken: for teaching hospitals, emergency, neonatal intensive care unit, thalassemia, surgery, and gynecology and obstetrics; for private hospitals, gynecology and obstetrics, coronary care unit, pediatrics, internal, and surgery; for social welfare hospitals, gynecology and obstetrics, surgery, internal, and intensive care unit; for the charity hospital, orthopedics, neonatal intensive care unit, gynecology and obstetrics, and surgery; and for the military hospital, surgery and internal. For all hospitals and wards, clinical use of blood and blood components and amount of discarded blood components were calculated. Blood wastage may occur for a number of reasons: time expiry (the expiry date on the unit has passed), wasted import (unit imported with patient but not used for storage condition reasons or poor practice at ward level and product discarded prior to the expiry date), blood medically or surgically ordered but not used (unit ordered for medical procedure but not necessary for treatment), no need for patient (nonmedical reasons such as death of patient, patient discharged, or no consent), leakage (damage to or fault in the blood bag), lack of cord blood (with unnecessary cross-matching for every medical procedure is thus impossible to re-do and blood is not used), hemolytic reasons, or miscellaneous reasons (any other reason for which the unit is wasted) (Table 2). The research instrument was a 2-part questionnaire. The first part was related to demographic characteristics of the hospitals and the second part elicited information about blood and blood component wastage; the data were taken from detailed forms of unused blood and blood components, categorized into 6 sections: type of blood product, blood groups, the number of requested units, the number of blood transfusion units, the number of wasted blood units, and the reason for wastage. Thus, the evaluation was retrospective. In order to complete the questionnaire, data were gathered by contacting the blood banks of the hospitals under study, conducting interviews with the people in charge of those departments, and studying the patients’ documents. The collected data were then analyzed using descriptive statistical methods and SPSS 11.5. 

## RESULTS

**Figure 1:**
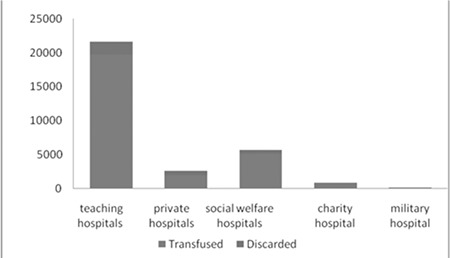
Total blood and blood product units transfused and discarded in hospitals.

**Figure 2:**
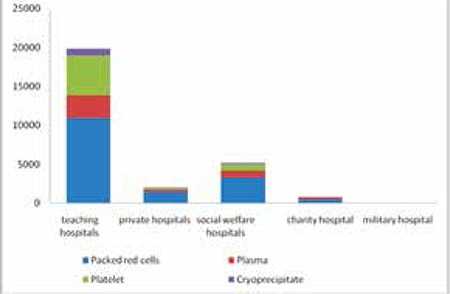
Total units transfused by type of blood and blood product in hospitals.

Out of a total of 30.913 units of blood received in all the hospitals of Qazvin Province, 58.6% were in the form of packed red cells, 0.3% in the form of whole blood, 15.8% in the form of plasma, 21.6% in the form of platelets, and 3.5% in the form of cryoprecipitate. Out of 3048 units of blood wasted, 59.4% were in the form of packed red cells, 22% in the form of plasma, 16% in the form of platelets, and 2.4% in the form of cryoprecipitate (Figures 1 and 2).

The distribution frequency of blood and blood product wastage in the hospitals of this study showed that out of a total of 3048 discarded units in all hospitals, 58.3% was wasted in teaching hospitals, 19.9% was wasted in private hospitals, 15.8% was wasted in social welfare hospitals, 3.2% was wasted in the charity hospital, and 1.9% was wasted in the military hospital. Teaching hospitals had the highest percentage of total blood and blood product waste, while private hospitals ranked second (Table 3). Results of packed red cell distribution frequency in different wards of teaching, private, social welfare, charity, and military hospitals indicated that the thalassemia ward had the highest use of packed red cells among different wards of teaching hospitals. The surgery ward had the highest use of packed red cells in the private and social welfare hospitals of Qazvin Province, while it was the gynecology and obstetrics ward that had the highest packed red cell use in the charity hospital. It should also be noted that the blood bank of the military hospital was not active and only 4 units out of its 62 packed red cell units were used in its internal medicine ward. 

Results of studying the packed red cell wastes in these hospitals showed that out of a total of 3048 discarded units in all hospitals, 1790 units belonged to teaching hospitals and 57.3% of these units were packed red cells, with the B+ blood type as the most often discarded one. Out of a total of 613 wasted units in private hospitals, 63.1% of waste also comprised packed red cells, while this was 56.5% of a total of 486 wasted units in social welfare hospitals, 64% of a total of 100 wasted units in the charity hospital, and 100% in the military hospital. 

Results of investigating the common causes of blood and blood product wastage in the hospitals of this study showed that blood and blood product wastes were highly associated with 3 common causes, including the expiration of the usability period, the patient’s lack of need for these products, and their non-use in hospital wards (Table 2).

## DISCUSSION

While the blood supply is seriously limited, blood use increases every year. Many of the blood transfusion cases are being done improperly. Proper clinical use of blood is an important and critical issue, especially in regions that are facing limitations in the number of blood units. Out of a total of 30.913 units received in all the hospitals of Qazvin Province in this study, 18.123 (58.6%) were in the form of packed red cells, 100 (0.3%) were in the form of whole blood, 4913 (15.8%) were in the form of plasma, 6695 (21.6%) were in the form of platelets, and 1082 (3.5%) were in the form of cryoprecipitate. The number of plasma units received during another study was 595 [15]. In another study on the use of fresh frozen plasma products at a children’s medical center, 1262 plasma units were received [16]. The current study saw a greater number of received units of blood and blood products from a larger study population. This study investigated the causes and the amount of blood wastes in all hospitals of Qazvin Province. It showed that blood and blood product wastes were highly associated with 3 common causes, including the expiration of the usability period, the patient’s lack of need for these products, and their non-use in hospital wards. There are a few reports about the average amount of expired blood units in blood banks or blood transfusion centers. In this study, 1410 (77.9%) out of a total of 1811 discarded packed red cell units were wasted due to the expiration of their usability period. In another study of decrease of blood waste using the sigma method, investigation of packed red cell wastes in hospitals showed that 87% of these wastes occurred outside the blood bank due to improper shipping and storage temperatures. Other factors involved were the personnel’s lack of knowledge and training, improper management of shipping temperature and other temperature indices, and the lack of supervision and responsibility [17]. The current study investigated the wastage of blood and blood products by hospitals of Qazvin Province. Results showed that the wastes were in the form of packed red cells (59.4%), plasma (22%), platelets (16%), and cryoprecipitate (2.4%) according to type of blood product, and out of a total of 3048 blood and blood product units wasted, 58.7% of them were returned by teaching hospitals, 20.1% by private hospitals, 15.9% by social welfare hospitals, 3.2% by the charity hospital, and 1.9% by the military hospital. A study reviewing blood transfusion and wastes in the teaching hospitals of Semnan University of Medical Sciences reported that 759 (65.9%) out of 1152 packed red cell units delivered to these hospitals were discarded and only 393 (34.1%) were transfused [18]. There are no reports that compare these amounts based on the type of hospitals. In another study of the use of blood and its products at Imam Reza Hospital, out of a total of 12.436 units of blood received by the blood bank, 2950 (23.8%) were discarded [19]. Results of studying the packed red cell wastes in the hospitals of the current study showed that out of a total of 3048 discarded units in all hospitals, 1790 units belonged to teaching hospitals and 57.3% of these units were packed red cells, with the B+ blood type as the most often discarded one. There are no similar studies that review cases of blood and blood product wastes based on different blood types. The thalassemia ward had the highest use of packed red cells among various wards of teaching hospitals in this study, while the emergency ward ranked second. In another study of unnecessary transfusion of blood and its products in hospitalized patients at Dr. Fatemi Hospital in Ardabil, 60 cases (40%) of blood transfusions occurred in the intensive care unit [20]. Blood transfusion and the supplying of healthy blood are among the more expensive medical services and most developing countries are not able to offer such services. Developing the proper culture for the optimum use of blood products in such countries is therefore of utmost importance. The current study recommends the following of some necessary policies for supervision and management of blood use and reduction of blood wastes, including monitoring consumers’ access to the supply of products, supervising the quality of new guidelines, investigating the reports received from hospitals regarding issues related to blood transfusion, inspecting the method of use of blood or its products in hospital wards, controlling the method of shipping from delivery to the hospitals until distribution among wards, and checking the hospital’s stock of blood and plasma in terms of numbers, dates, method of storage, and the time of keeping blood for cross-matching. The hemovigilance system is being used in 2 teaching hospitals of Qazvin Province to reduce blood wastes and it allows the possibility of promoting the blood transfusion chain (from the time of blood collection until the time of its injection into the recipient) and monitoring the quality of blood and its products’ use, better so than before. According to the recommendations of the World Health Organization, blood transfusion should be prescribed only in situations where other methods cannot effectively prevent or control mortality. Decisions about the prescription of blood must be done based on the most reliable clinical guidelines available, which are modified according to the needs of patients. Blood products have a limited half-life, so the approach to blood transfusion must apply accurate strategies for blood reserves in order to prevent loss and reduce wastes as much as possible [21]. In overall evaluation of hospitals, considering the ratio of waste to usage, it should be noted that teaching hospitals had the best practice and military hospital had the worst practice. This study addresses one of the research priorities of Iran’s blood transfusion system. The advantages of this research are that it included all the hospitals in Qazvin Province and that a large number of reasons for blood and blood product wastage were considered. Of course, it is the first comprehensive study that was done in Iran, while other studies regarding these issues were not as comprehensive as this research as they did not include all the hospitals and only considered 1 or 2 blood products. It is hoped that this research will help to decrease the amount of blood wastage, as blood is an important and rare resource. 

## ACKNOWLEDGMENTS

This article was extracted from a thesis that was supported financially by the School of Health, Qazvin University of Medical Sciences. We thank all who assisted us in the process of developing this study, with special thanks to the blood banks of the hospitals and personnel in charge of these departments for their strong support and high commitment during the preparation of this survey. 

## CONFLICT OF INTEREST STATEMENT

The authors of this paper have no conflicts of interest, including specific financial interests, relationships, and/or affiliations relevant to the subject matter or materials included. 

## Figures and Tables

**Table 1:**
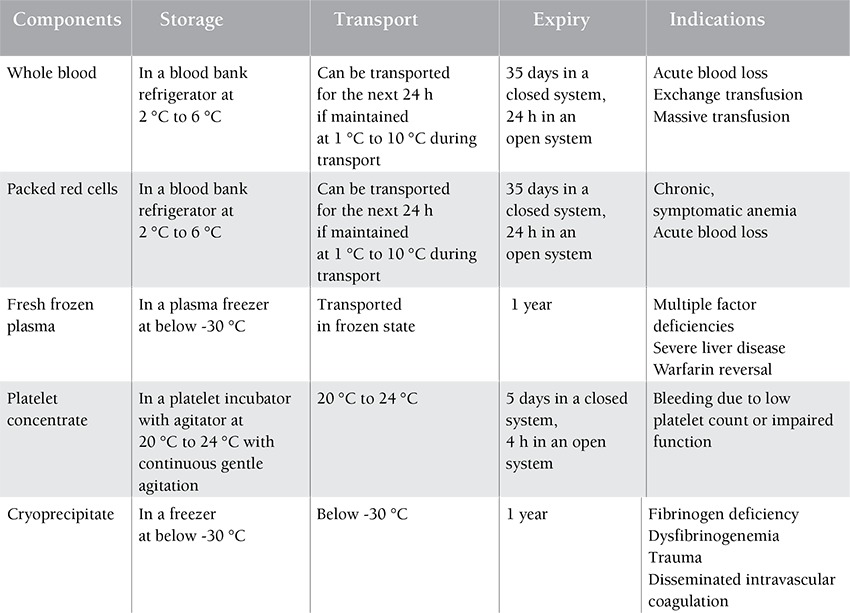
Requirements for storage and transport, expiration, and indications of blood and blood components.

**Table 2:**
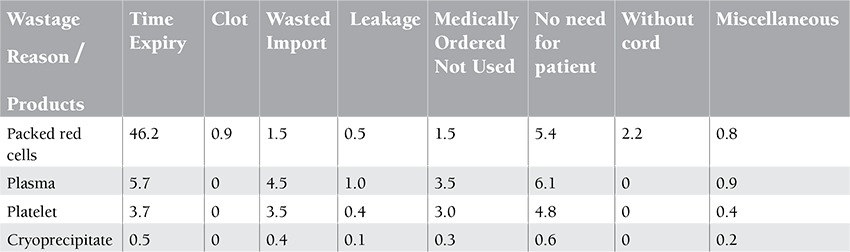
Percentage of wastage per cause of discard in total hospitals.

**Table 3:**
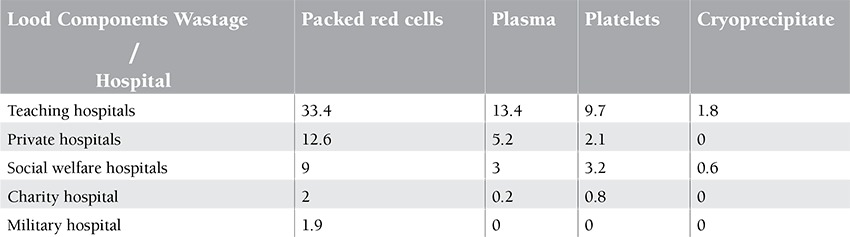
Percentage of wastage by type of blood and blood product in hospitals.
